# Neutrophil extracellular traps (NETs) reduce the diffusion of doxorubicin which may attenuate its ability to induce apoptosis of ovarian cancer cells

**DOI:** 10.1016/j.heliyon.2022.e09730

**Published:** 2022-06-15

**Authors:** Kohei Tamura, Hideyo Miyato, Rihito Kanamaru, Ai Sadatomo, Kazuya Takahashi, Hideyuki Ohzawa, Takahiro Koyanagi, Yasushi Saga, Yuji Takei, Hiroyuki Fujiwara, Alan Kawarai Lefor, Naohiro Sata, Joji Kitayama

**Affiliations:** a Department of Obstetrics and Gynecology, Jichi Medical University, Shimotsuke, Japan; b Department of Surgery, Jichi Medical University, Shimotsuke, Japan; c Department of Clinical Oncology, Jichi Medical University, Shimotsuke, Japan; d Center for Clinical Research, Jichi Medical University Hospital, Shimotsuke, Japan

**Keywords:** Neutrophil extracellular traps, Doxorubicin, Chemosensitivity, Pharmacokinetics

## Abstract

**Purpose:**

Although neutrophil extracellular traps (NETs) are present in various tumors, their roles in tumor biology have not been clarified yet. In this study, we examined how NETs affect the pharmacokinetics and effects of doxorubicin (DOX).

**Methods:**

NETs were generated by neutrophils stimulated with phorbol 12-myristate 13-acetate (PMA) or lipopolysaccharide (LPS). DOX was added to NETs and their distribution was observed under fluorescein microscopy, and the diffusion of DOX through 3 μM pores from lower to upper chambers was evaluated with a fluorescence-based assay. Ovarian cancer cells, KOC-2S and SKOV3, were embedded in collagen gel droplets and cultured in 3D way and their apoptosis was examined with flow cytometry.

**Results:**

DOX was mostly co-localized with NETs. The transfer of DOX to upper chambers increased over time, which was significantly decreased by the presence of neutrophils stimulated with PMA or LPS in the lower chamber. DOX outside of the gel increased the rates of annexin V (+) apoptotic cells, which were significantly reduced by the addition of LPS-stimulated neutrophils in media both in KOC-2S and SKOV3. The reduced diffusion and apoptosis were mostly restored by the destruction of the NETs structure with 1000 u/ml DNAse I.

**Conclusion:**

NETs efficiently trap and inhibit the diffusion of DOX which may attenuate its ability to induce apoptosis of ovarian cancer cells. Degradation of NETs with DNAse I may augment the response of ovarian cancer to DOX.

## Highlights

• Doxorubicin is efficiently trapped by neutrophil extracellular traps (NETs).
• NETs suppress diffusion of doxorubicin through micro-pores and infiltration into resected tumor.
• NETs suppress doxorubicin-induced apoptosis of tumor cells in 3-D culture.
• DNAse may augment the effect of anti-cancer drugs by modulating pharmacokinetics.

## 1 Introduction

Neutrophils are the most abundant cell type among circulating leukocytes and kill microbes through a variety of mechanisms, such as phagocytosis, the release of reactive oxygen species (ROS) and cytosolic enzymes, and neutrophil extracellular traps (NETs) [1]. It is well known that neutrophils are also present in many different types of malignancies and are referred to as tumor-associated neutrophils (TAN) [2, 3]. Recently, many immunohistochemical studies using specific antibodies to Citrullinated histones 3 (cit-H3) as well as CD15 or CD66b, have suggested that neutrophils infiltrating human and murine tumors form substantial amounts of NETs [4, 5, 6, 7, 8].

NETs are complexes of chromosomal DNA, histones, and granule proteins released by activated neutrophils which ensnare extracellular microbes [9]. Emerging evidence suggests that NETs might play important roles in various noninfectious diseases, including autoimmune diseases, thrombosis and cancer [10, 11]. In particular, NETs have been shown to have tumor-promoting functions, such as sequestration of circulating tumor cells causing metastases [7, 12], induction of epithelial mesenchymal transition (EMT) [13] and creation of a tumor permissive microenvironment [8, 14]. Other studies have shown that NETs can also trap and degrade cytokines and chemokines which limits inflammation in gout [15, 16]. However, how NETs in tumor microenvironments interact with anti-cancer drugs and modulate their effects remains unclarified. In this study, we asked whether NETs could affect the pharmacokinetics and apoptotic effects of the widely used chemotherapeutic agent, doxorubicin (DOX).

## 2 Materials and methods

### 2.1 Reagents and cells

Doxorubicin (DOX) (≥98%), a crystalline solid, was purchased from Cayman Chemical (Ann Arbor, MI) and dissolved with DMSO at a concentration of 10 mg/ml and stored at -80 °C before the use in experiments. Phorbol 12-myristate 13-acetate (PMA) (≥97%) and lipopolysaccharide (LPS) (≥97%) from Wako Pure Chemical (Osaka, Japan) and Sigma (St. Louis, MO), respectively. SYTOX green nucleic acid stain (≥99%) and DNAse I (≥90%) were purchased from Thermo Fisher Scientific (Waltham, MA) and Worthington Biochemical Co. (Lakewood, NJ), respectively. FITC-conjugated Annexin V and 7-AAD were from BioLegend (San Diego, CA) and Thermo Fisher Scientific, respectively. The human ovarian serous adenocarcinoma cell lines SKOV-3 was obtained from American Type Culture Collection (ATCC) and maintained in Dulbecco’s Modified Eagle Medium (DMEM) supplemented with 10% fetal bovine serum (FBS; Sigma, St. Louis, MO), 100 U/mL penicillin, and 100 mg/mL streptomycin (Life Technologies, Grand Island, NY). The KOC-2S cell line which was established from a poorly differentiated serous ovarian adenocarcinoma in 1993 [17] was given by Dr. Kataoka (Kurume University), and expanded for 3 passages in the same medium and used for experiments.

### 2.2 Neutrophil purification and production of NETs

Peripheral blood was obtained from healthy volunteers with permission. After dextran sedimentation, leukocyte-enriched plasma was overlaid on Ficoll-Hypaque solution (Cytiva, Uppsala, Sweden) and centrifuged at 3000 rpm for 15 min. The bottom layers were taken and washed twice with PBS + 0.02% EDTA. Red blood cells (RBC) were removed with RBC lysis buffer (BioLegend, San Diego, CA) according to the manufacturer’ recommendation and washed twice with PBS + 0.02% EDTA. The fractions of polymorphonuclear leukocytes (PMN) containing ≥95% neutrophils were used for the following experiments.

NETs were produced by the rolling method as described previously [18]. Briefly, isolated neutrophils (5 × 10^6^/ml) were stimulated with 2 μM PMA or 10 μg/ml lipopolysaccharide in RPMI 1640 medium and the tubes rotated at 37 °C for 30 min using a tube roller device (Miltenyi Biotec, Bergisch Gladbach, Germany) to avoid cell clumping. The cells were washed three times with PBS + 0.02% EDTA to fully remove PMA or LPS, and the stimulated cells resuspended in HBSS without phenol red (Nacalai Tesque, Kyoto, Japan) and cultured on poly-L-lysine-coated 6-well plates for an additional 4 h in a humidified tissue culture incubator at 37 °C, 5% CO_2_ atmosphere. In all experiments, NET formation was confirmed by the addition of 50nM SYTOX Green (Thermo Fisher Scientific, Waltham, MA) to bind extracellular DNA components. As a control, neutrophils were pre-incubated with vehicle and placed at 4 °C for 4 h.

### 2.3 Fluorescein microscopy

Neutrophils (5 × 10^6^) were stimulated with PMA or LPS were cultured in 6 well plates for 4 h and DOX added at a final concentration of 20 μM. After 30 min incubation, the wells were gently washed with warmed media and SYTOX green was added at a concentration of 50 nM. DOX and NETs structures were observed using a fluorescence microscope (BZ8000, Keyence, Osaka, Japan) under optical wavelength filters for Tetramethylrhodamine (TRICI) and FITC, respectively. Finally, the distributions of DOX and NETs were evaluated with the superimposed images taken using appropriate wavelength filters.

### 2.4 Diffusion of drugs with NETs

The effect of NETs on diffusion of anti-cancer drugs was examined with a double chamber system consisting of 3 μM pore trans well inserts (Corning Inc., Corning, NY, USA) and polystyrene 6-well plate (Thermo Fisher Scientific, Waltham, MA). In brief, neutrophils (5 × 10^6^) stimulated with PMA or LPS as described above were resuspended in 2 mL of HBSS without phenol red, placed in the bottom chambers and cultured for 4 h to form NETs. DOX (20 μM) was added to the bottom chambers and incubated for another 30 min. Then, culture inserts with pores containing 2 mL of colorless HBSS were placed in the bottom chambers. After the indicated number of hours, 100 μl of fluid from the upper chambers were collected and auto fluorescence intensities of DOX measured using a microplate plate reader (Berthold Technologies, Wildbad, Germany) with excitation/emission wavelengths of 485/535 nm. In some experiments, NETs were pre-incubated with 1000 u/ml DNAse I for 30 min to degrade NETs before addition of the DOX.

### 2.5 DOX infiltration in excised tumor

Peritoneal tumors were induced by intraperitoneal injection of an ovarian cancer cell line, SKOV-3 (1 × 106), in balb/c nude mice. After 3 weeks, similar sized tumors (approximately 3∼5 mm in diameters) were resected and soaked in 50 mM DOX diluted in 4 ml of HBSS buffer with unstimulated or PMA-stimulated neutrophils in 15 ml tubes. DNase I (1000 u/ml) was added with PMA-stimulated neutrophils at the start of the experiment. After 3 h, the tumors were removed, fixed with dry-ice/acetone and 10-μM cryostat sections of post-fixed frozen samples were created. After the counterstaining nuclei with DAPI, the infiltration of DOX from the tumor surface was evaluated by detection of autofluorescence under fluorescence microscopy (BZ8000; Keyence, Osaka, Japan).

### 2.6 Apoptosis assay in collagen gel droplet culture

Collagen gel droplet-embedded culture drug sensitivity tests were performed as described previously [19]. In brief, Type I collagen, Ham’s F-12 medium at 10-fold concentration and reconstitution buffer (Nitta Gelatin., Osaka, Japan) were mixed at a ratio of 8:1:1 on ice. KOC-2S or SKOV3 (1 × 10^6^) cells were suspended in 3 ml of the cold solution. These collagen mixtures were dropped into 6 well plates as 5 spots each with a volume of 30 μl and subjected to dome-like gelation at 37 °C in 5% CO_2_ for 1 h. Then, the droplets were cultured in DMEM supplemented with 2% FBS with or without 15 μM DOX. Unstimulated or activated neutrophils with LPS (1 × 10^7^/2 ml) were added in DMEM with or without DNAse I (1000 U/ml). After 12 h, collagen gels were digested with 0.02% collagenase type 1 (Thermo Fisher Scientific, Waltham, MA) and cells recovered. After washing, the ratios of apoptosis were evaluated using flow cytometry FACSCalibur (BD Biosciences, Franklin Lakes, NJ). In brief, all cells were incubated with FITC-conjugated Annexin V and 7-AAD at a final concentrations of 2.25 μg/ml and 0.1 μg/ml, respectively, for 15 min at 4 °C. In flow cytometric profile (FSCxSCC), tumor cells were gated and the ratios of annexin V (+) cells were calculated in the gated area.

### 2.7 Statistical analysis

Data are shown as mean ± standard deviation (SD) and compared using one-way ANOVA followed by Tukey’s honestly significant difference tests. P values <0.05 were considered statistically significant.

## 3 Results

### 3.1 DOX is trapped by NETs

**Figure 1:**
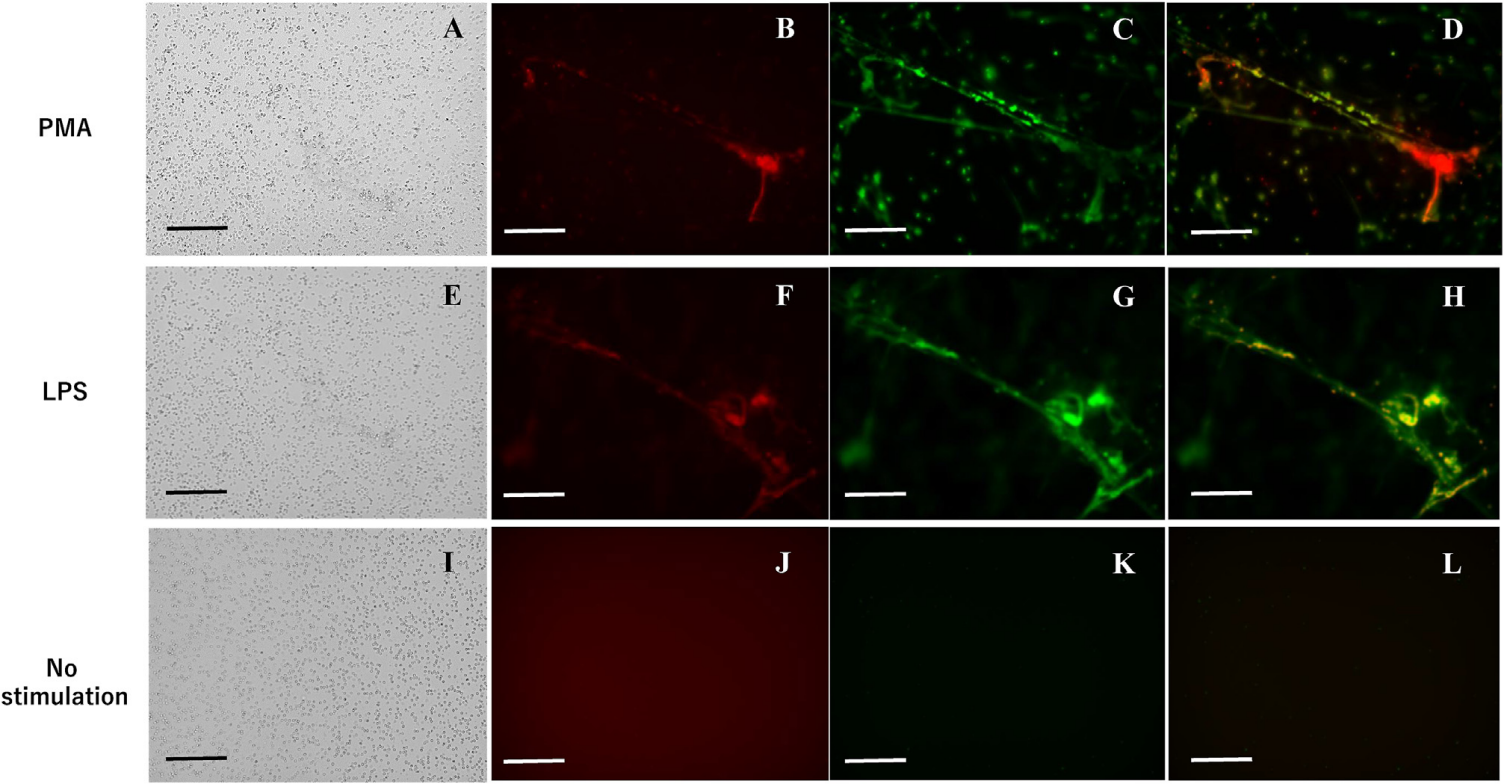
Fluorescein images of doxorubicin (DOX) and neutrophil extracellular traps (NETs). NETs were generated by stimulating neutrophils with 2 μM PMA (A–D) or 10 μg/ml LPS (E–H) and cultured in poly-L-lysine-coated plates as described in Materials and Methods. Then, DOX was added at a final concentration of 20 μM and incubated with NETs for 30 min. After gentle washing with warmed media, SYTOX green (50 nM) was added. As controls, DOX was added to freshly isolated neutrophils without washing (I–L). Images of bright fields (A, E, I). DOX was visualized using fluorescence microscopy with the optical wavelength filter for Tetramethyl rhodamine (TRICI) (B, F, J). NETs structures in the same field were observed with a wavelength filter for FITC (C, G, K) and the 2 fluorescein images were superimposed (D, H, L). Bars show a length of 100 μM.

First, NETs were generated by stimulating purified neutrophils with 2 μM PMA and their physical interaction with DOX was examined. After co-incubation with stimulated neutrophils for 30 min and gentle washing, many neutrophils remained attached to the poly-L-lysine-coated plate (Figure 1A), and red auto fluorescence of DOX was detected in a specific area (Figure 1B). When NETs were visualized with SYTOX green, red DOX was mostly merged in the green area of NET structures (Figure 1B, C ,D). Since PMA stimulation is not physiologic, we next used LPS. Preincubation of neutrophils with 10 μg/ml LPS for the initial 30 min was enough to stimulate the production of large amounts of NETs. DOX was similarly co-localized with these NETs (Figure 1E, F, G, H). However, when DOX was incubated with fresh neutrophils for 30 min, NETs were not detected, and DOX was evenly distributed in media (Figure 1J, K, L). This indicates that DOX is preferentially bound by NETs regardless of the method of stimulation. Similar colocalization was detected between Oregon Green-conjugated paclitaxel (PTX) and SYTOX orange-stained NETs (Supplementary Figure 1A).

### 3.2 Diffusion of DOX is inhibited by activated neutrophils, but not by unstimulated neutrophils

**Figure 2:**
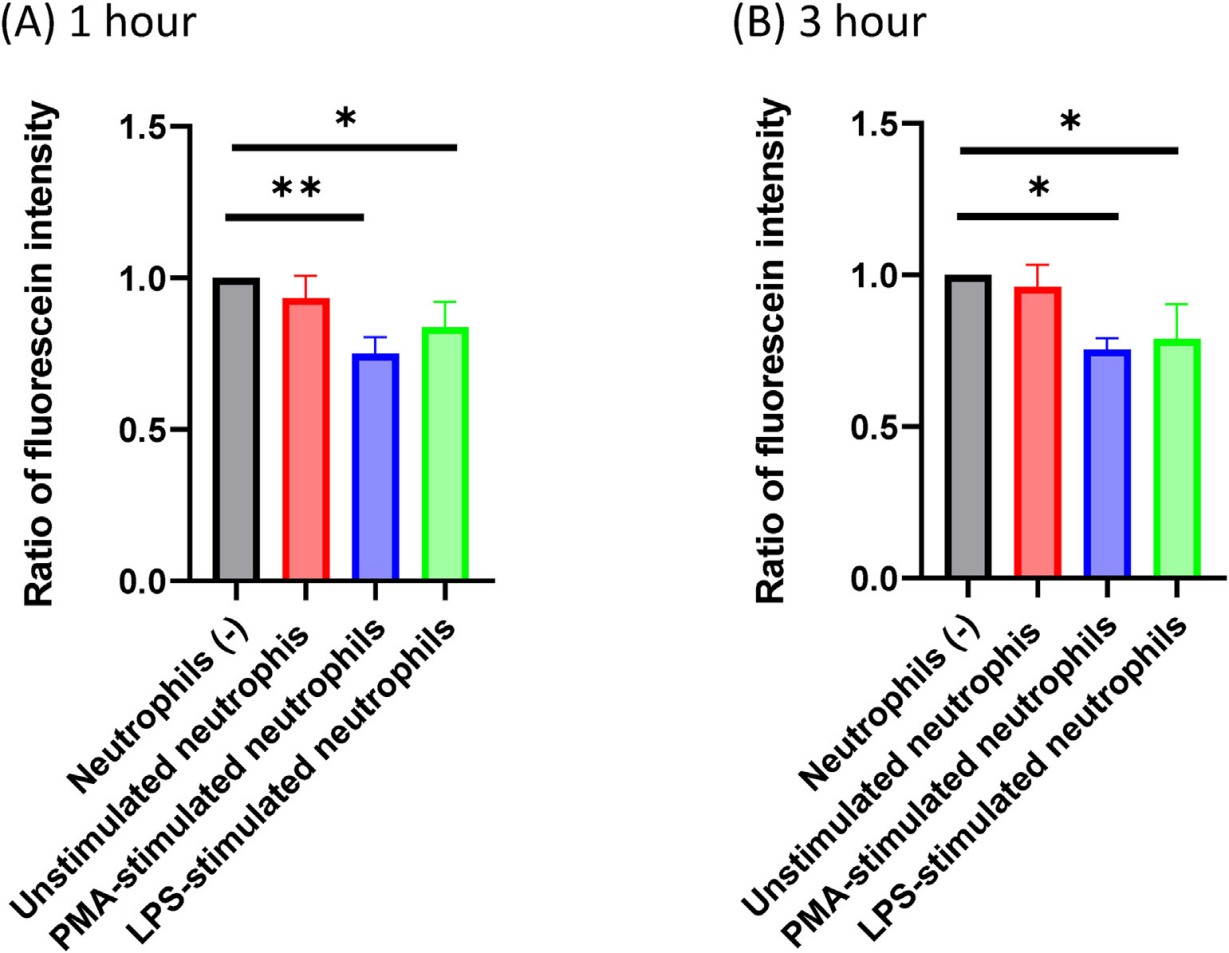
Diffusion of doxorubicin (DOX) through culture inserts with 3 μM pores. Neutrophils (5 × 10^6^) stimulated with 2 μM PMA or 10 μg/ml LPS were suspended in 2 mL of HBSS without phenol red and placed in the bottom chambers and cultured for 4 h to form NETs. Freshly isolated neutrophils (5 × 10^6^/2 mL) were placed at 4 °C and used as unstimulated neutrophils. DOX (10 μM) was added and incubated for another 30 min. Then, culture inserts with pores containing 2 mL of HBSS were placed in the bottom chambers. After 1 (A) and 3 (B) hours, 100 μl of medium was collected from the upper chambers and fluorescence intensity measured. The relative ratios of the fluorescein intensities were calculated compared to the control well which did not contain neutrophils. Data are shown as mean ± standard deviation in 3 different experiments. ∗: p < 0.05, ∗∗: p < 0.01.

The diffusion efficiency of DOX was examined with a double chamber system though a 3 μM pore membrane. To avoid the physical effects of closing small pores by NET structures, DOX was placed in the lower chamber with or without neutrophils and then the diffusion of DOX was evaluated by measuring auto fluorescence intensities of fluid in the upper chamber. As shown in Figure 2 when PMA-stimulated neutrophils were placed in the lower chamber, the fluorescence intensities in the upper chamber were reduced by 20–31% at 1 (p = 0.0049) and 3 h (p = 0.011). Similarly, the presence of LPS-stimulated neutrophils significantly reduced the fluorescence intensities in the upper chamber (p = 0.050 at 1 h, p = 0.026 at 3 h). However, when unstimulated neutrophils were placed in lower chamber, the fluorescence intensities were not significantly altered at any time point.

### 3.3 NETs inhibit the diffusion of DOX

**Figure 3:**
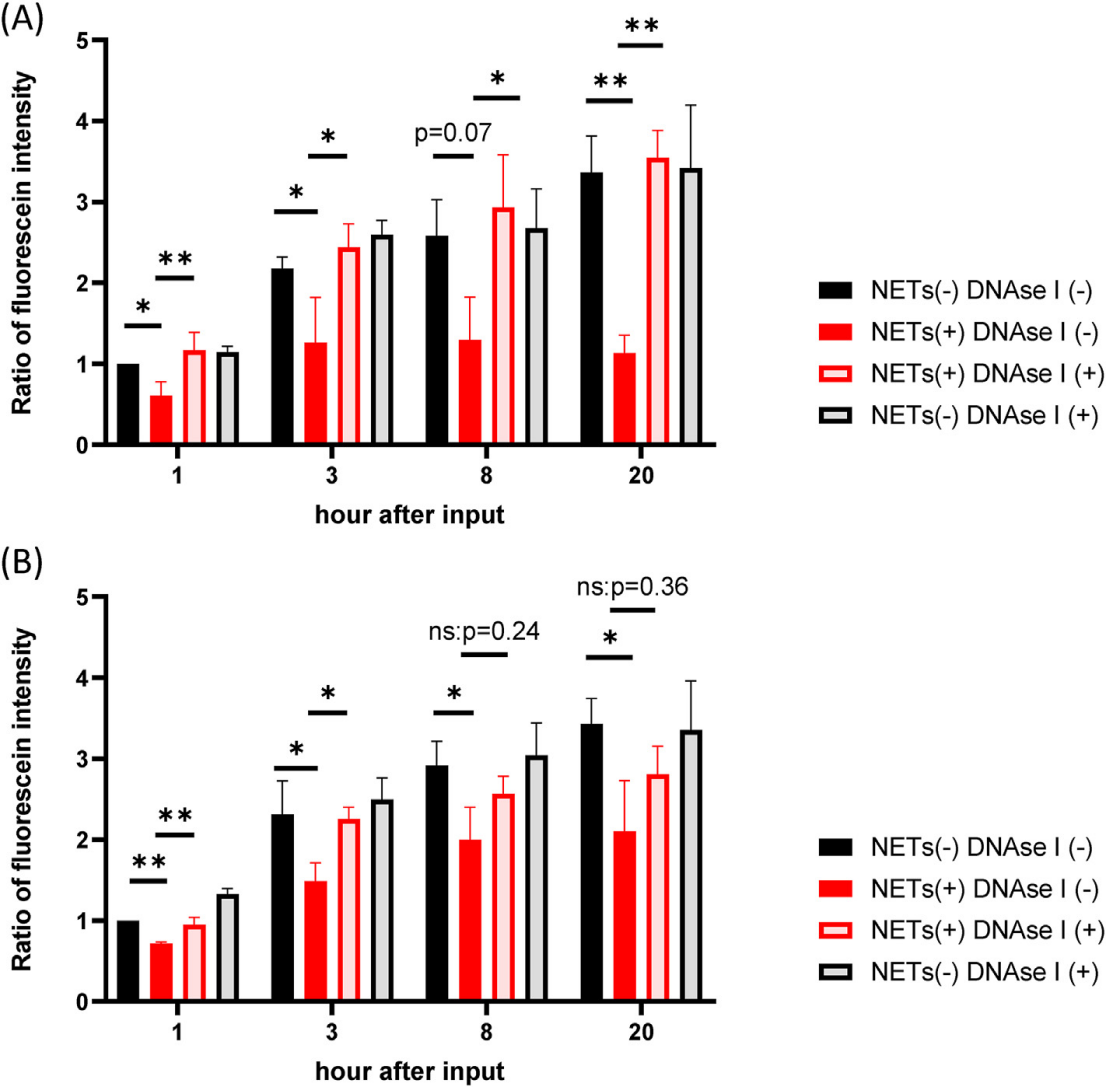
The effects of NETs on the diffusion of doxorubicin (DOX). Neutrophils (1 × 10^7^/4 ml) stimulated with PMA (A) or LPS (B) were placed in the bottom chamber as described in Figure 2 legend and DOX added at a final concentration of 10 μM and incubated for another 30 min. In some wells, DNAse I was added at a final concentration of 1000 u/ml at 30 min before the addition of DOX. Then, culture inserts containing 2 mL of HBSS were placed in the bottom chambers and auto fluorescence intensities of DOX in the upper chamber measured at the indicated time points. In each set of experiments, the relative ratios of fluorescein intensities were calculated against the value of the samples measured at 1 h after incubation in control wells which did not contain NETs and DNAse I. Data are shown as mean ± standard deviation in 3 (PMA) and 3 (LPS) different experiments. ∗: p < 0.05, ∗∗: p < 0.01.

Whether the effect on DOX diffusion is dependent on NETs produced by PMA-stimulated neutrophils was then examined. As shown in Figure 3A, fluorescence intensities of media from the upper chambers increased until 20 h. The fluorescence intensity at each time point was reduced by the presence of activated neutrophils by 39–66% with significant differences at 1 (p = 0.043), 3 (p = 0.041), 8 (p = 0.070) and 20 h (p = 0.0025). However, when 1000 u/ml DNAse I was added in the lower chamber to degrade NETs, the intensities in the upper chamber were restored to the same levels as controls at all time points (p = 0.0068 at 1 h, p = 0.011 at 3 h, p = 0.023 at 8 h, p < 0.0015 at 20 h).

When DOX was placed with LPS-stimulated PMN, the fluorescence intensities in the upper chamber also decreased 28–39% over time (p = 0.0013 at 1 h, p = 0.028 at 3hr. p = 0.041 at 8 h., p = 0.044 at 20 h) (Figure 3B). Fluorescence intensities were recovered by the presence of DNAse I, although the effects was less prominent compared with PMA-stimulated neutrophils (p = 0.0050 at 1 h, p = 0.040 at 3 h) and not significant at later time points. These observations suggest that the inhibition of DOX diffusion is mostly dependent on capture by NETs in the lower chamber.

The diffusion of Oregon green conjugated paclitaxel was also examined with same method (Supplementary Figure 1B). Similar to changes seen in the presence of DOX, the fluorescein intensities in the upper chamber were reduced by the presence of PMA-stimulated neutrophils. However, the reduced fluorescein intensities were not restored by pretreatment of the activated neutrophils with DNAse I.

### 3.4 NETs inhibit the infiltration of DOX in excised tumor

**Figure 4:**
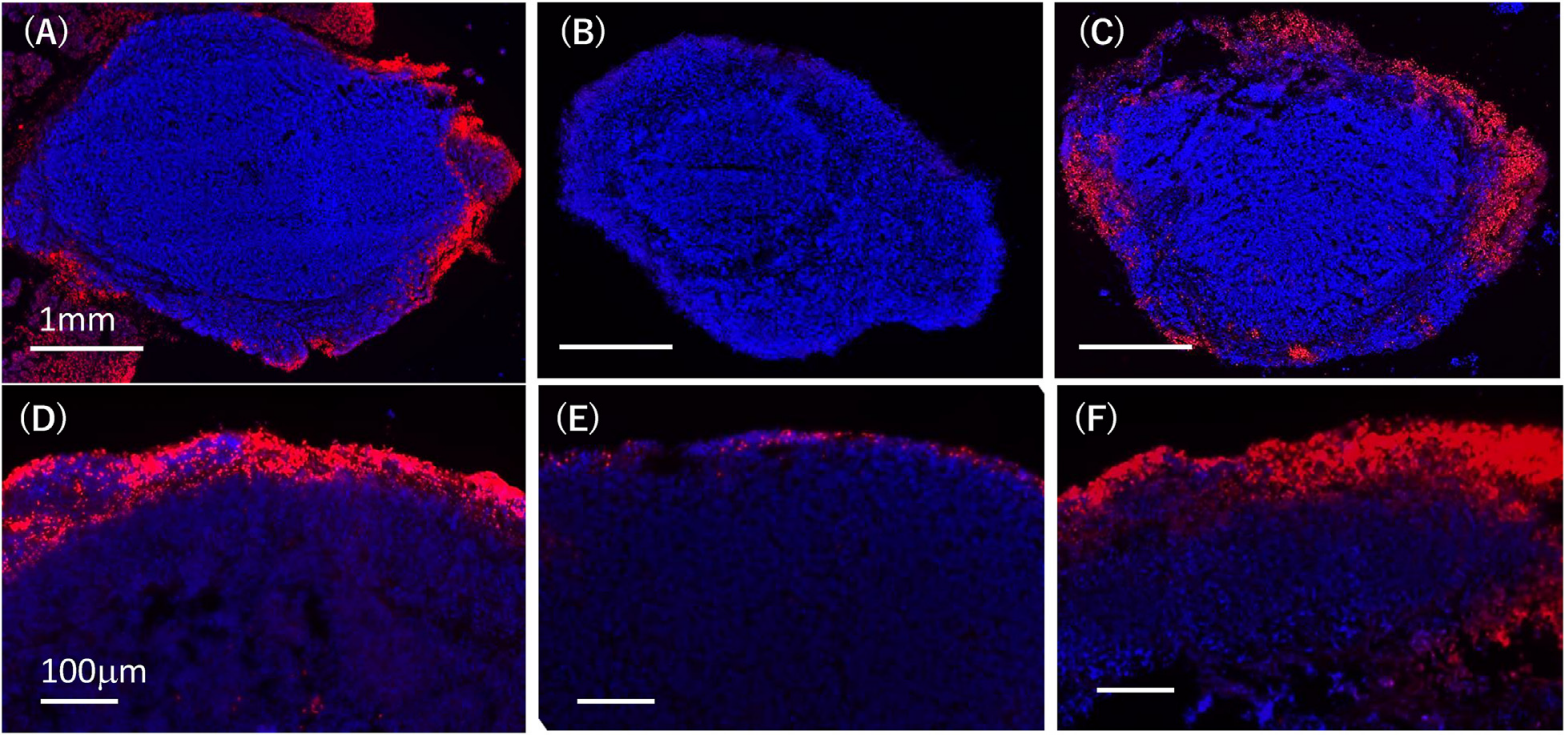
Peritoneal tumors of SKOV-3 were induced as described in Materials and Methods, and similar sized tumors (approximately 3∼5 mm in diameters) were soaked in 50 mM DOX diluted in 4 ml of HBSS buffer with unstimulated (A) or PMA-stimulated (B) neutrophils in 15 ml tube. In (C), DNase I (1000 u/ml) was added with PMA-stimulated neutrophils at the start of the experiment. After 3 h, the tumors were taken out, fixed with dry-iced acetone and 10-μM cryostat sections of post-fixed frozen samples were created. After the counterstaining the nuclei with DAPI, the infiltration of DOX from the tumor surface was evaluated with the detection of autofluorescence under fluorescence microscopy (BZ8000; Keyence, Osaka, Japan). Figures show the merged images for DOX (red) and DAPI (Blue).

We next examined whether DOX infiltration was altered by the NETs in an *ex vivo* model. Peritoneal tumors were induced by intraperitoneal injection of an ovarian cancer cell line, SKOV-3, in nude mice and resected after 2 weeks. Then, tumors of similar size were soaked in 50 mM DOX diluted in 4 ml of HBSS buffer containing unstimulated or PMA-stimulated neutrophils. After 3 h, diffusion of DOX was determined with frozen section samples using a fluorescence microscope (Figure 4). The penetration of DOX from the tumor surface was significantly impaired by the presence of PMA-stimulated neutrophils compared with unstimulated neutrophils, and the impaired penetration was restored by the degradation of NETs by DNAse I.

### 3.5 NETs attenuate DOX-induced apoptosis of ovarian cancer cells in 3-dimensional culture

Next, whether NETs affect the apoptosis of tumor cells induced by DOX in a 3-D culture system was evaluated using a collagen gel droplet assay (Supplementary Figure 2A). When KOC–S2 cells embedded in collagen cell were cultured with PMA-stimulated neutrophils for 20 h, the number of apoptotic cells detected with positive staining by annexin V increased by 4–5 times probably because of the excessive amounts of cytotoxic substances released from PMA-activated neutrophils. However, LPS-stimulated neutrophils did not significantly affect the ratio of annexin V (+) KOC-S2 and thus LPS-stimulated neutrophils were used in the following experiments (Supplementary Figure 2B).

**Figure 5:**
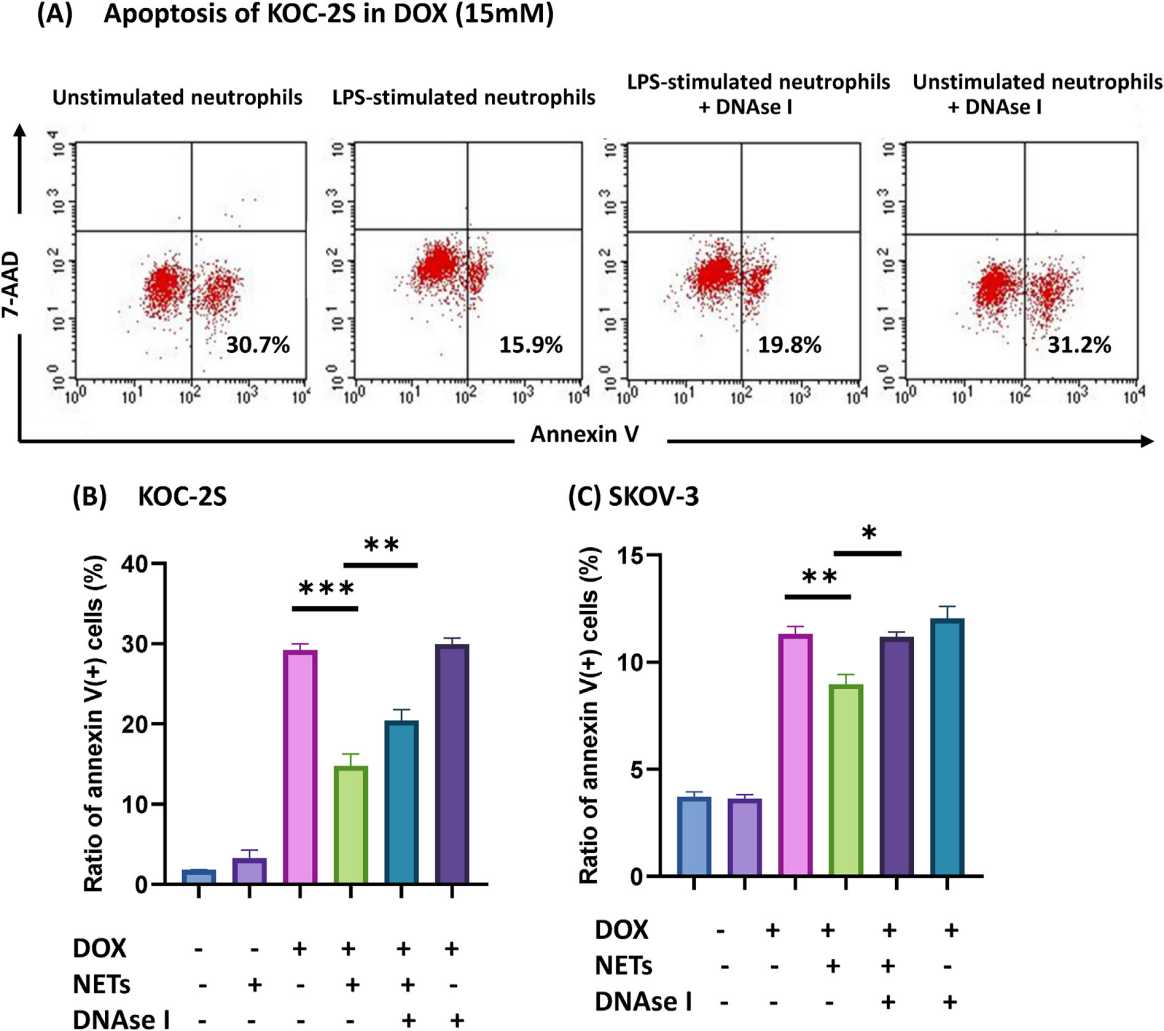
KOC-2S or SKOV3 cells were embedded in collagen gel droplets as described in Materials and Methods and cultured in 2 ml media containing 15 μM doxorubicin (DOX) with neutrophils (1 × 10^7^) and/or DNAse I (1000U/ml). NETs (-) and (+) show the data in the presence of unstimulated and LPS-stimulated neutrophils, respectively. After 12 h, apoptotic cells were examined with FACSCalibur. (A) Representative FACS Profiles of KOC-2S (B) Data are shown as mean ± standard deviation in triplicate from one of 4 (KOC-2S) and 3 (SCOV-3) different experiments. ∗: p < 0.05, ∗∗: p < 0.01, ∗∗∗: p < 0.001.

As shown in Figure 5 A and B, when collagen droplets were incubated with DOX and unstimulated neutrophils for 12 h at a final concentration 15 μM, many KOC-S2 cells showed apoptosis detected by annexin V (29.2 ± 0.75%). When LPS-stimulated neutrophils were present together with DOX, the ratios of annexin V (+) cells were obviously decreased to 14.8 ± 1.44% (p < 0.0001). However, if NETs were degraded by 1000 u/ml DNAse I, the rate of apoptosis was significantly increased (20.4 ± 1.37%, p = 0.002). Similarly, DOX induced apoptosis in 11.3 ± 0.36% of SKOV-3 cells, which was significantly decreased in the presence of NETs derived from LPS-activated neutrophils (9.0 ± 0.46%, p = 0.003). The inhibitory effects on apoptosis were abrogated by the presence of DNAse I (10.9 ± 0.64, p = 0.013) (Figure 5C).

## 4 Discussion

NETs are structures composed of a DNA scaffold with associated with histones and cytosolic proteases which are released by activated neutrophils [9, 20]. An increasing number of studies have suggested that NETs are positively involved in cancer metastasis and relapse [7, 12, 21] as well as cancer associated thrombosis [22] and tumor immunoediting [4]. However, the role of NETs in the tumor microenvironment has not completely elucidated.

In this study, we found that DOX, one of the most frequently used anti-cancer drugs, is preferentially accumulated at the area of NETs generated with PMA- or LPS-stimulated neutrophils, and its diffusion through micro-pores is significantly decreased by the presence of NETs. Since DOX fluorescence might be affected by pH, we measured the fluorescein intensities of 1 mM and 5 mM DOX dissolved in HBSS at various pH conditions. However, the fluorescein intensities of DOX did not show significant differences within the range of pH under the conditions used in these experiments (6.83–7.42), although the intensity of 5 mM DOX was significantly elevated at pH 5.6 (Supplementary Figure 3). This indicates that the phenomenon is not related to the changes in pH caused by the addition of NETs. Moreover, the reduced diffusion was mostly restored by degradation of the NETs with DNAse I. The same finding was observed in ex vivo experiments using peritoneal tumors. These observations suggest that NETs efficiently trap DOX, which hinders the availability of the drug. Since DOX has a high DNA binding affinity and inhibits the growth of malignant cells though the inhibition of DNA synthesis [23, 24], this is not surprising but appears to be reasonable. The phenomenon was less prominent when NETs were obtained by the stimulation of neutrophils with LPS compared with PMA. In fact, some LPS-stimulated neutrophils underwent necrosis instead of NETosis, which may be the reason why treatment with DNAse I does not fully restore the reduced diffusion of DOX after a longer time interval.

As shown in Supplementary Figure 1, we found that NETs can also trap and inhibit the diffusion of Oregon green-conjugated paclitaxel. However, the reduced diffusion was not restored by the degradation of NETs with DNAse I. Paclitaxel is known to inhibit the cell growth thorough the induction of tubulin polymerization and stabilization against their depolymerization [25]. Therefore, it is speculated that PTX bound to polymerized tubulin still remains as large molecular complexes and may not diffuse through the micropores, even when DNA fibers were broken into small pieces by DNAse I.

More importantly, the presence of LPS-stimulated neutrophils in culture media also suppressed apoptosis of tumor cells induced by DOX in 3D culture, and the reduced apoptosis was also restored by treatment with DNAse I. NETs might have direct effects on tumor cell apoptosis. Then, we examined whether NETs exist in the collagen gels using SYTOX green. As shown in Supplementary Figure 4, NETs were detected in the media outside of the gels, while no staining was detected in collagen gels at cell recovery, indicating that NETs cannot invade into the gels as they are. Therefore, it is unlikely that NETs have direct effects on tumor cell apoptosis. Although *in vivo* data are necessary, these observations suggest the possibility that NETs in tumors or surrounding tissues may efficiently trap DOX and attenuate its anti-tumor effects by interfering with rapid and uniform drug distribution in the tumor.

In this study, we examined the diffusion of DOX at a high concentration (10–15 μM) due to the sensitivity of the fluorescence-based assay. The concentrations of DOX are higher than the peak concentration of doxorubicin in serum in patients with cancer who received a systemic infusion [26]. However, DOX is often used as an intravesical injection for bladder cancer [27] or intraperitoneal administration for peritoneal metastases [28]. Since DOX is often given at higher concentrations for those patients, the results of this study are clinically relevant, at least, in cases of local treatment.

NETs can also be detected in human serum as double strand-DNA combined with myeloperoxidase (MPO) by ELISA and their levels are reported to be elevated in patients with various inflammatory diseases such as acute lung injury [29], sepsis [30] and Covid-19 infection [31] as well as thrombosis [32]. Moreover, Zhang et al recently reported that the serum level of NETs is inversely correlated with short-term efficacy in patients with advanced gastric cancer who received first-line chemotherapy agents [14]. Given that NETs can also bind systemically administered DOX in circulating blood, NETs in serum may significantly impair the pharmacokinetics of systemically administrated drugs. Indeed, recent studies have suggested the possibility that NETs may have a role in resistance to chemo-, immuno- or radiotherapy [33].

In summary, NETs trap and suppress the diffusion of DOX and attenuate its effects to induce apoptosis of tumor cells. To the best of our knowledge, this is the first report to show a direct interaction between anticancer drugs and NETs. In addition to DOX and paclitaxel, NETs may be able to capture other anti-cancer drugs, especially drugs with high DNA binding affinity such as mitomycin C (MMC) or platinum preparations. Pharmacological interference with NET formation or destruction of NETs may enhance the therapeutic effects of these drugs. Park et al. have reported that DNAse I-coated nanoparticles reduce lung metastases in a murine model [34]. The local delivery of such long acting DNAse I is an intriguing strategy to improve the sensitivity of tumor cells to chemotherapeutic agents, especially in patients with tumors containing large numbers of NETs.

## Declarations

### Author contribution statement

Kohei Tamura; Hideyo Miyato; Rihito Kanamaru; Ai Sadatomo: Conceived and designed the experiments; Performed the experiments.

Kazuya Takahashi; Hideyuki Ohzawa: Performed the experiments; Analysed and interpreted the data.

Takahiro Koyanagi; Yasushi Saga; Yuji Takei: Contributed reagents, materials, analysis tools or data.

Hiroyuki Fujiwara: Conceived and designed the experiments; Contributed reagents, materials, analysis tools or data.

Alan Kawarai Lefor; Naohiro Sata: Contributed reagents, materials, analysis tools or data; wrote the paper.

Joji Kitayama: Conceived and designed the experiments; analysed and interpreted the data; wrote the paper.

### Funding statement

This work was supported by Grant-in-Aid for Scientific Research from 10.13039/501100001691Japan Society for the Promotion of Science (20K22960 and 21K16432) and 10.13039/501100005865Mochida Memorial Foundation for Medical and Pharmaceutical Research.

### Data availability statement

All data generated or analysed during this study are included in this published article and its supplementary information files.

### Declaration of interest’s statement

The authors declare no conflict of interest.

### Additional information

No additional information is available for this paper.

## Appendix A Supplementary data

Supplementary Figure v4 **Supplemental Figure 1:** (A) Fluorescein images of paclitaxel and neutrophil extracellular traps (NETs). NETs were generated by stimulating neutrophils with 2μM PMA and Oregon green-conjugated paclitaxel were added at a final concentration of 0.2mM and the distribution was observed under fluorescence microscopy. NETs were visualized with SYTOX orange (50nM). Paclitaxel images were superimposed with bright field images (upper) and NETs images (lower). Bar shows 100mm. (B) Diffusion of Paclitaxel. Oregon green-conjugated paclitaxel was placed in the lower chamber at a final concentration of 2mM in the presence or absence of neutrophils (5x106) stimulated with 2μM PMA and/or 1000u/ml DNAse I. Then, culture inserts with 3mm pores containing 2mL of HBSS were placed in the bottom chambers. After 1∼20 hours, 100μl of medium was collected from the upper chambers and fluorescence intensity measured. Data are shown as mean ± standard deviation in triplicate from one of the 3 different experiments. **Supplemental Figure 2:** Apoptosis assay with collagen gel droplet-embedded culture. (A) KOC-2S cells (1×10^6^) were embedded in collagen gel and dropped onto 6 well plates as 5 spots each with a volume of 30μl as described in Materials and Methods. (B) The droplets were filled with DMEM supplemented with 2% FBS with or without PMA- or LPS-stimulated neutrophils (5x106). After 20 hours, the wells were washed, and the collagen gel digested with collagenase. Recovered cells were stained with FITC-conjugated annexin V and 7-AAD. In the tumor cell population gated in the flowcytometric profile (FSCxSCC), ratios of annexin V (+) apoptotic cells were calculated. Values shown are the percentages of annexin V (+) tumor cells. **Supplemental Figure 3:** pH of HBSS without phenol red was adjusted to 5.6 to 7.6 using 1mol/L hydrochloric acid. DOX was dissolved in the media at concentrations of 1mM and 5mM, and their fluorescein intensities were measured in quadruplicate. The p-value among the data were calculated with one way ANOVA. **Supplemental Figure 4:** Collagen gel droplets (30μl) were made on plastic plates as described in Materials and Methods, and PMA-stimulated neutrophils (1x10^7^) in 2ml of DMEM supplemented with 2% FBS were added. After 12 hours incubation, medium was removed, and NETs were visualized with SYTOX green (50nM) under the fluorescence microscope. Supplementary Figure v4
