# Supplementary material for: Neutrophil extracellular traps (NETs) reduce the diffusion of doxorubicin which may attenuate its ability to induce apoptosis of ovarian cancer cells
Source: Heliyon. 2022 Jun 15;8(6):e09730. doi: 10.1016/j.heliyon.2022.e09730 (PMC9218137; doi:10.1016/j.heliyon.2022.e09730)
Supplement: Supplementary Figure v4 — Supplemental Figure 1: (A) Fluorescein images of paclitaxel and neutrophil extracellular traps (NETs). NETs were generated by stimulating neutrophils with 2μM PMA and Oregon green-conjugated paclitaxel were added at a final concentration of 0.2mM and the distribution was observed under fluorescence microscopy. NETs were visualized with SYTOX orange (50nM). Paclitaxel images were superimposed with bright field images (upper) and NETs images (lower). Bar shows 100mm. (B) Diffusion of Paclitaxel. Oregon green-conjugated paclitaxel was placed in the lower chamber at a final concentration of 2mM in the presence or absence of neutrophils (5x10^6^) stimulated with 2μM PMA and/or 1000u/ml DNAse I. Then, culture inserts with 3mm pores containing 2mL of HBSS were placed in the bottom chambers. After 1∼20 hours, 100μl of medium was collected from the upper chambers and fluorescence intensity measured. Data are shown as mean ± standard deviation in triplicate from one of the 3 different experiments. Supplemental Figure 2: Apoptosis assay with collagen gel droplet-embedded culture. (A) KOC-2S cells (1×10^6^) were embedded in collagen gel and dropped onto 6 well plates as 5 spots each with a volume of 30μl as described in Materials and Methods. (B) The droplets were filled with DMEM supplemented with 2% FBS with or without PMA- or LPS-stimulated neutrophils (5x10^6^). After 20 hours, the wells were washed, and the collagen gel digested with collagenase. Recovered cells were stained with FITC-conjugated annexin V and 7-AAD. In the tumor cell population gated in the flowcytometric profile (FSCxSCC), ratios of annexin V (+) apoptotic cells were calculated. Values shown are the percentages of annexin V (+) tumor cells. Supplemental Figure 3: pH of HBSS without phenol red was adjusted to 5.6 to 7.6 using 1mol/L hydrochloric acid. DOX was dissolved in the media at concentrations of 1mM and 5mM, and their fluorescein intensities were measured in quadruplicate. The p-value among the data were [file mmc1.pptx]

## Slide 1
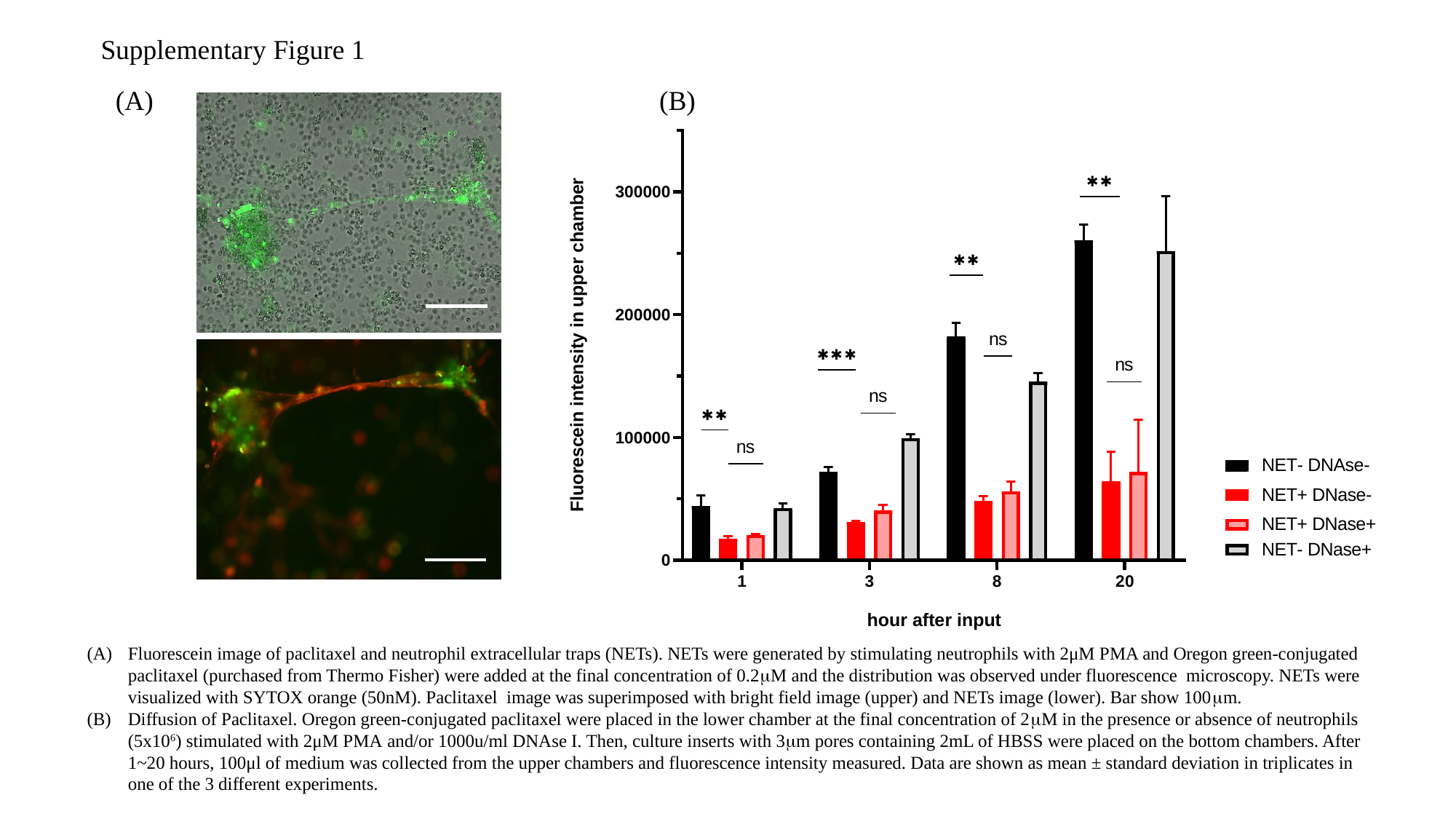

Supplementary Figure 1
(A)
(B)
Fluorescein image of paclitaxel and neutrophil extracellular traps (NETs). NETs were generated by stimulating neutrophils with 2μM PMA and Oregon green-conjugated paclitaxel (purchased from Thermo Fisher) were added at the final concentration of 0.2mM and the distribution was observed under fluorescence microscopy. NETs were visualized with SYTOX orange (50nM). Paclitaxel image was superimposed with bright field image (upper) and NETs image (lower). Bar show 100mm.
Diffusion of Paclitaxel. Oregon green-conjugated paclitaxel were placed in the lower chamber at the final concentration of 2mM in the presence or absence of neutrophils (5x10^6^) stimulated with 2μM PMA and/or 1000u/ml DNAse I. Then, culture inserts with 3mm pores containing 2mL of HBSS were placed on the bottom chambers. After 1~20 hours, 100μl of medium was collected from the upper chambers and fluorescence intensity measured. Data are shown as mean ± standard deviation in triplicates in one of the 3 different experiments.

## Slide 2
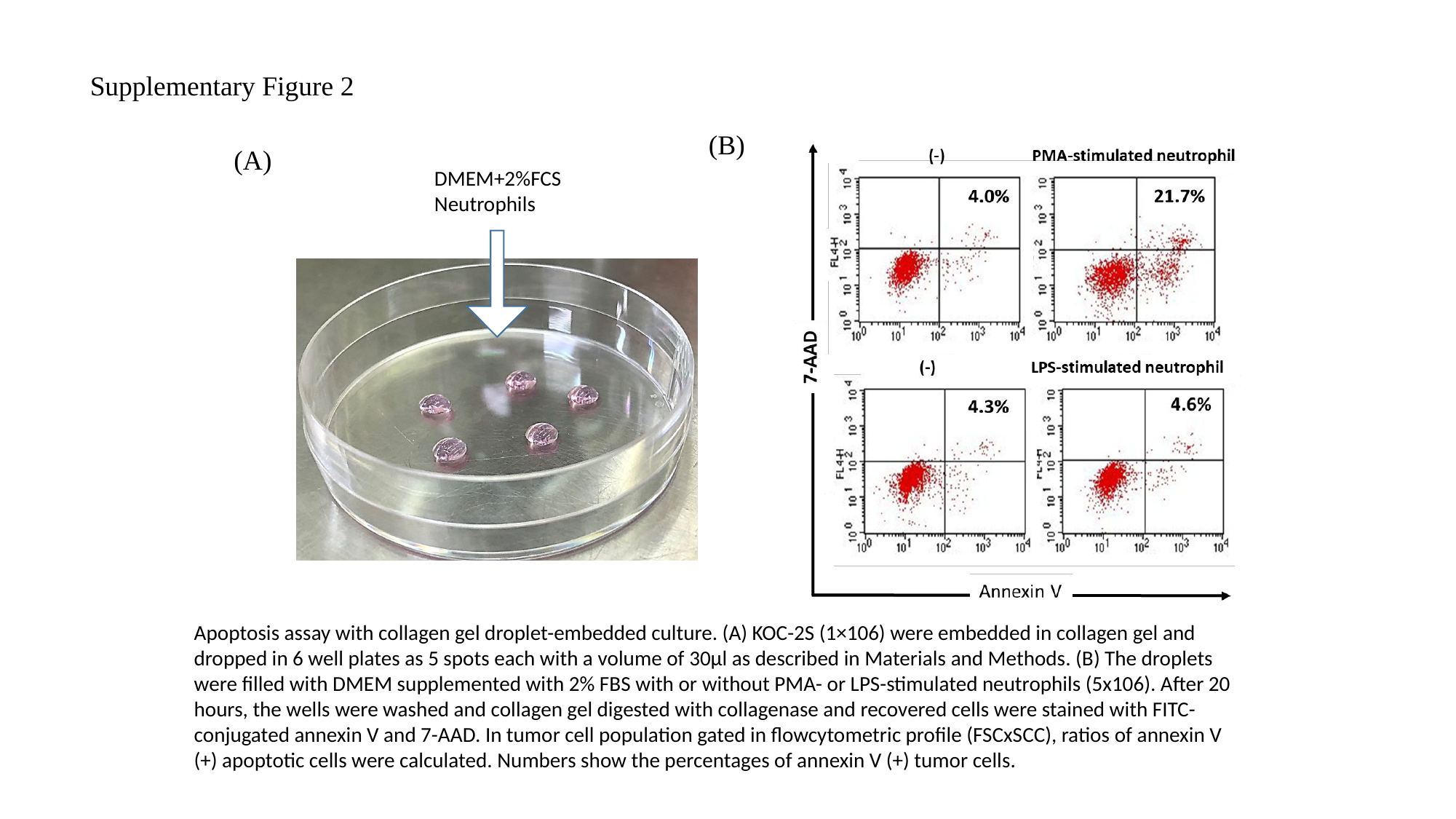

Supplementary Figure 2
(B)
(A)
DMEM+2%FCS
Neutrophils
Apoptosis assay with collagen gel droplet-embedded culture. (A) KOC-2S (1×10^6^) were embedded in collagen gel and dropped in 6 well plates as 5 spots each with a volume of 30μl as described in Materials and Methods. (B) The droplets were filled with DMEM supplemented with 2% FBS with or without PMA- or LPS-stimulated neutrophils (5x10^6^). After 20 hours, the wells were washed and collagen gel digested with collagenase and recovered cells were stained with FITC-conjugated annexin V and 7-AAD. In tumor cell population gated in flowcytometric profile (FSCxSCC), ratios of annexin V (+) apoptotic cells were calculated. Numbers show the percentages of annexin V (+) tumor cells.

## Slide 3
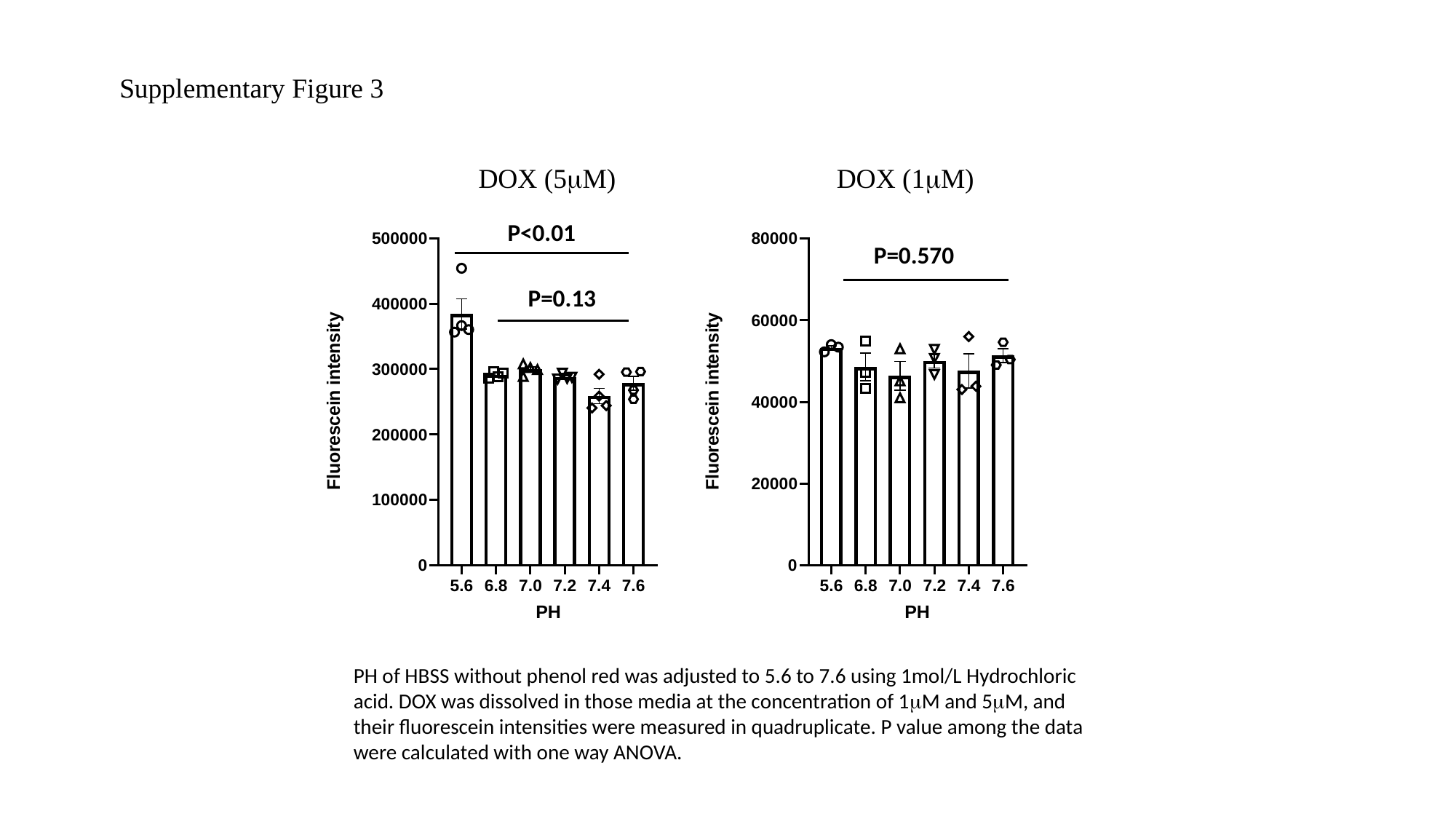

Supplementary Figure 3
DOX (5mM)
DOX (1mM)
P=0.570
P=0.13
P<0.01
PH of HBSS without phenol red was adjusted to 5.6 to 7.6 using 1mol/L Hydrochloric acid. DOX was dissolved in those media at the concentration of 1mM and 5mM, and their fluorescein intensities were measured in quadruplicate. P value among the data were calculated with one way ANOVA.

## Slide 4
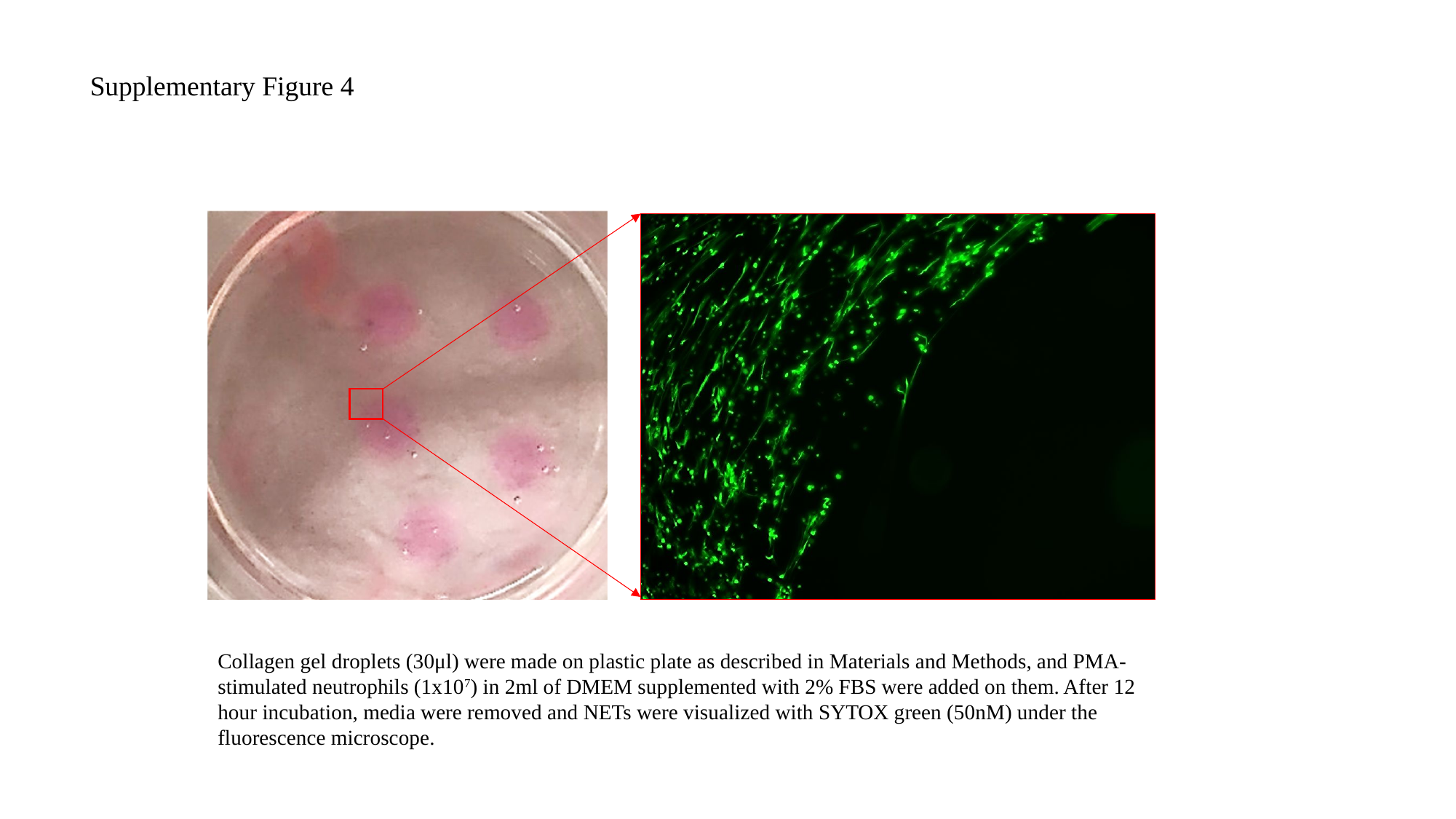

Supplementary Figure 4
Collagen gel droplets (30μl) were made on plastic plate as described in Materials and Methods, and PMA-stimulated neutrophils (1x10^7^) in 2ml of DMEM supplemented with 2% FBS were added on them. After 12 hour incubation, media were removed and NETs were visualized with SYTOX green (50nM) under the fluorescence microscope.
